# Determinants of internal migrant health and the healthy migrant effect in South India: a mixed methods study

**DOI:** 10.1186/s12914-017-0132-4

**Published:** 2017-09-12

**Authors:** Warren Dodd, Sally Humphries, Kirit Patel, Shannon Majowicz, Matthew Little, Cate Dewey

**Affiliations:** 1 Department of Population Medicine, University of Guelph, Guelph, ON N1G 2W1 Canada; 2 Department of Sociology and Anthropology, University of Guelph, Guelph, ON N1G 2W1 Canada; 3 International Development Studies Program, Menno Simons College affiliated with the University of Winnipeg and Canadian Mennonite University, Winnipeg, MB R3C 0G2 Canada; 4 School of Public Health and Health Systems, University of Waterloo, Waterloo, Ontario N2L 3G1 Canada

**Keywords:** India, Migration, Migrant health, Occupational health, Health status, Determinants of health

## Abstract

**Background:**

Internal labour migration is an important and necessary livelihood strategy for millions of individuals and households in India. However, the precarious position of migrant workers within Indian society may have consequences for the health of these individuals. Previous research on the connections between health and labour mobility within India have primarily focused on the negative health outcomes associated with this practice. Thus, there is a need to better identify the determinants of internal migrant health and how these determinants shape migrant health outcomes.

**Methods:**

An exploratory mixed methods study was conducted in 26 villages in the Krishnagiri district of Tamil Nadu. Sixty-six semi-structured interviews were completed using snowball sampling, followed by 300 household surveys using multi-stage random sampling. For qualitative data, an analysis of themes and content was completed. For quantitative data, information on current participation in internal labour migration, in addition to self-reported morbidity and determinants of internal migrant health, was collected. Morbidity categories were compared between migrant and non-migrant adults (age 14–65 years) using a Fisher’s exact test.

**Results:**

Of the 300 households surveyed, 137 households (45.7%) had at least one current migrant member, with 205 migrant and 1012 non-migrant adults (age 14–65 years) included in this study.

The health profile of migrant and non-migrants was similar in this setting, with 53 migrants (25.9%) currently suffering from a health problem compared to 273 non-migrants (27.0%). Migrant households identified both occupational and livelihood factors that contributed to changes in the health of their migrant members. These determinants of internal migrant health were corroborated and further expanded on through the semi-structured interviews.

**Conclusions:**

Internal labour migration in and of itself is not a determinant of health, as participation in labour mobility can contribute to an improvement in health, a decline in health, or no change in health among migrant workers. Targeted public health interventions should focus on addressing the determinants of internal migrant health to enhance the contributions these individuals can make to their households and villages of origin.

## Background

Internal labour migration is a necessary livelihood strategy for millions of individuals and households throughout India. According to an examination of official employment statistics from industries that predominantly employ migrant workers, it is estimated that at there are at least 100 million internal migrant workers in the country [1]. With all of these individuals and families engaged in temporary or permanent movements for work, there is optimism surrounding the ability of internal labour migration to meaningfully contribute to human development [2]. In particular, there are often economic incentives associated with migrating for work, which can contribute to material gain for migrants and their families [2, 3]. In addition, the relative stability and frequency of financial transfers between migrants and their families, as well as the circulation of ideas, knowledge, and technology, can extend the economic benefits of migrant labour to households and communities of origin [3–6]. However, there is also broad recognition that participation in labour mobility may have significant consequences for the health and wellbeing of migrant workers, and these consequences may pose challenges in leveraging the purported benefits of internal labour migration [3, 6, 7].

Previous research on the relationship between health and internal labour migration in India has primarily examined the negative health outcomes among migrant workers as a result of their participation in labour mobility. Disease transmission among migrant workers, and between migrant workers and their households and communities of origin, is a prominent area of focus, with specific interest in HIV and AIDS, in addition to other sexually transmitted infections [8–12]. Additional studies have examined health outcomes associated with hazardous workplace conditions experienced by migrant workers in specific industries such as textile factories [13, 14], manual labour [15, 16], and construction [17–19]. There has also been evidence to suggest that the prevalence of mental health problems is higher among migrant individuals than non-migrants [20]. In terms of non-communicable and chronic disease, migrants may be at an elevated risk for obesity [21]. In addition, participation in migration may be associated with some negative changes in dietary habits including the higher consumption of energy and fat [22]. Conversely, there has been some research into the broad health benefits experienced by households of origin attributed to internal migration [23], yet research on any potential health benefits attributed to internal labour migration for migrant workers in India is limited [see 22 for improvements in dietary diversity].

In addition to research on health outcomes, other studies have focused on the vulnerability of migrant workers in India and how this vulnerability shapes migrant worker experiences with health. In particular, reference is made to the ‘invisibility’ of migrants within Indian social policy leading to barriers in accessing services including healthcare [24]. Poor health outcomes among migrant workers are further exacerbated because of these barriers, and as a result, improvements in migrant worker healthcare access combined with targeted preventive public health initiatives are considered to concretely improve migrant worker health outcomes and reduce vulnerability [2, 3, 25].

Despite this vulnerability, there is also recognition that participation in internal labour migration is a selective process whereby migrant workers may have a health advantage over their non-migrant counterparts [26]. This health advantage is the premise of the ‘healthy migrant effect’, which is the notion that migrant workers have better than expected health outcomes when the socioeconomic conditions of their place of origin are taken into account [27]. Although typically applied to international migration, there is some evidence to suggest the presence of the ‘healthy migrant effect’ among internal migrant workers in Croatia [28], Indonesia [26], and China [29–31]. However, research on migration from low-income to high-income countries demonstrates that this potential health advantage is difficult to maintain over the long-term [32–34]. In particular, problems with poverty, housing, stress related to a new environment, nutrition, substance abuse and poor access to healthcare can contribute to the loss of this apparent health advantage [27].

Thus, there is a need for evidence-based public health policies and interventions that directly identify and address the determinants of internal migration health [25, 35, 36]. With this need in mind, and drawing on both qualitative and quantitative data, this study has two objectives: first, to explore if and how the ‘healthy migrant effect’ operates in this setting; and second, to identify the broad determinants of internal migrant health and to examine how these determinants influence the health of migrant workers.

## Methods

### Study area and design

This exploratory mixed method study was conducted in 26 rural villages in Anchetty, Thaggatti, Madakkal, and Urigam *panchayats* within the Krishnagiri district of Tamil Nadu, India. These adjoined *panchayats* are located in the northwest corner of Tamil Nadu in the Melagiri Hill Ranges of the Eastern Ghats along the border with Karnataka. While all of the *panchayats* have road access, Anchetty *panchayat* is the best networked in terms of quality of roads and access to public transportation, and the town of Anchetty proper is a regional market hub. Conversely, Urigam is the most geographically remote *panchayat*, and consequently, was only included in the qualitative portion of this study.

Rates of poverty and illiteracy are higher than district averages within the study site, with 36% of the population living below the poverty line and an adult literacy rate of 48.3% [37].^1^ Despite ongoing investment in agricultural and rural development, as well as access to social welfare schemes, the research area’s proximity to the urban centres of Bengaluru and Hosur means that internal labour migration is prevalent among households [38]. The economic incentives for migration are strong, with some migrants reportedly earning double or triple the amount of income per day through migrant labour compared to local employment opportunities. Adult males from large families and historically marginalized castes (Schedule Castes, Scheduled Tribes, Other Backwards Castes, Most Backwards Castes) were the most likely to participate in labour migration from the research area. Labour migration was largely temporary in nature, meaning that a typical migration trip lasted 1 week to 6 months. Additionally, rural to urban labour migration comprised the majority of movements, although there was some rural to rural migration originating from this area.

This study was part of a larger interdisciplinary international development research project titled ‘Revalorizing Small Millets in the Rainfed Regions of South Asia’ (RESMISA). Based in Nepal, India, and Sri Lanka, the RESMISA project received funding between 2011 and 2014 through the Canadian International Food Security Research Fund (CIFSRF) delivered by the International Development Research Centre (IDRC) and the Department of Foreign Affairs, Trade and Development (DFATD; formerly, the Canadian International Development Agency and now Global Affairs Canada). Locally, the study detailed here received support and guidance from Development of Humane Action (DHAN) Foundation, which was a leading non-governmental organization within the RESMISA project. As an organization with expertise in agricultural and community development, in addition to a field office in the town of Anchetty proper, DHAN Foundation contextualized the research questions and approach and assisted in terms of obtaining the necessary local research approvals.

### Qualitative methods and analysis

A semi-structured interview guide exploring health and migration dynamics was developed with input from DHAN Foundation and informed by community engagement in the research villages. In particular, the interview guide inquired about common illnesses and the general course of treatment for each illness. In December 2012, 66 semi-structured interviews were completed with 66 different households using snowball sampling in 17 villages within the four *panchayats* included in the study. With translation assistance, each interview was conducted in Tamil or Kannada according to the respondent’s preference. In cases where two individuals actively participated in the interview process through consulting each other prior to responding to a question (often a male and female couple), quotations were attributed to both individuals involved. Interviews were audio recorded and the research assistant translated each response into English. Interviews were manually transcribed and any discrepancies in translation were clarified with the research assistant. An analysis of themes and content was completed and informed the subsequent survey development (below).

### Quantitative methods and analysis

Based on the preliminary analysis of the semi-structured interviews, a survey tool was developed to collect information on socioeconomic status, labour migration, and health. To inquire about self-reported morbidity, a list of common illnesses was compiled that were identified through the interviews for inclusion with the survey. The survey was piloted and refined with four individuals from four different households in two different villages in Anchetty *panchayat*, and any questions that posed problems were clarified. Then, between January–March 2013, 300 household surveys were completed in 20 rural villages in Anchetty, Thaggatti, and Madakkal *panchayats*.

Multistage random sampling was used to sample villages and then households within villages. Approximately half of the villages within each *panchayat* were randomly sampled and included in the study. Then, approximately 10% (8.1%–12.7%) of households within each village were systematically randomly sampled (approximately every tenth household was included) based on the estimated number of households. The female or male household head was surveyed and served as a proxy respondent for all household members. Only adults (≥18 years) were asked to act as the proxy respondent. Virilocal families living within one housing structure were considered as a single household unit. Surveys were delivered in either Tamil or Kannada depending on the respondent’s preference, and responses were recorded directly onto the questionnaire in English. As part of survey administration, each respondent was verbally provided with the previously generated list of common illnesses as a prompt, and then asked if any member of their household was currently suffering from any of these illnesses. At the end of each day of survey administration, the first author reviewed the questionnaires with the research assistant to ensure completeness and to clarify information.

To achieve equal representation of both females and males in the study, survey administration was timed to avoid interfering with women’s daily household responsibilities. Additionally, if present in the household, an adult female was first asked to participate in the study and only if she was unable or unwilling to participate, was a male member of the same household asked to participate. The response rate was 96.5%, with 300 of 314 households completing the survey. Non-response was attributed to lack of interest.

To assess participation in labour migration, a migrant was defined as an individual who was engaged in labour outside of her or his village of usual residence at the time of survey administration as reported by the survey respondent. In addition, the skill level of different occupations was self-defined by survey respondents and based on the amount training or education required for each position. Self-reported illnesses were initially recorded using the respondents’ lay terms and later categorized according to the International Classification of Diseases’ (ICD) version 10 broad categories [39] with adaptations according to local context [40].

All data from the surveys was entered and managed in Microsoft Excel® by the first author and validated by a research assistant. Data were analyzed using Stata® 12 statistical package. The prevalence of each self-reported illness category was compared between migrant and non-migrant adults (age 14–65). Data were stratified based on sex and age. For age, the median age of 40 years was used to create two groups (i.e., < 40 years and ≥ 40 years). A Fisher’s exact test was used to assess if there was a difference in the prevalence of the various disease categories between migrants and non-migrants for each sex and age strata.

### Ethical considerations

Ethics clearance was obtained from a Canadian university research ethics board. In India, permission was obtained from local authorities to conduct the study including from panchayat councils, government medical staff, and law enforcement. As previously indicated, the study was also supported and sponsored by a national non-governmental organization called DHAN Foundation that reviewed and approved the study. Informed oral consent was obtained to prior to each interview or survey. This process involved each respondent receiving a detailed explanation of the study and having the opportunity to ask questions. If a potential respondent had no desire to participate for any reason, the research team continued to the next nearest household.

## Results

### Socio-demographic information of study participants

Of the 66 semi-structured interviews, 53 (80.3%) were completed with a male respondent. The average age of interviewees was 48 years (24–75 years). Interviewees had an average of 3.6 years (0–12 years) of formal education, with 26 interviewees (39.4%) having no formal education. The average household size among interviewees was 5.7 members (1–15 members), and 49 respondents (86.0%) were members of households where subsistence agriculture was an important livelihood strategy. In total, 35 (53.0%) interviewees had at least one member from their household engaged in migration at the time of the interview, with 50 migrant workers included across these 35 households. Of these 50 current migrant workers, 49 were male (98.0%). The average age of all migrant workers included in the qualitative part of the study was 27.4 years (17–56 years).

Of the 300 households surveyed, 137 households (45.7%) had at least one current migrant member with 205 migrant workers included in this study. In total, 188 migrants (91.7%) were male and the average age of all migrant workers was 27.5 years (14–65 years; SD = 8.44) compared to 34.2 years (SD = 14.71) for non-migrant adults in the same age cohort (*p* < 0.001). Most migrants included in this study were engaged in rural to urban migration streams with many (73.7%) working in the nearby urban centres of either Bengaluru or Hosur. In addition, most migrants were engaged in either low skilled (131 individuals; 63.9%) or semi-skilled work (55 individuals; 26.8%). The main industries that migrants were working in included construction (92 individuals; 44.9%), manual labour (33 individuals; 16.1%), the textile sector (18 individuals; 8.8%), and manufacturing (15 individuals; 7.3%). Detailed results on both the determinants and outcomes of labour migration in this setting can be found in our previous studies [41, 42].

### Self-reported health outcomes

Based on the household surveys, 53 migrants (25.9%) were currently suffering from a health problem. Table 1 provides an overview of all health events experienced by adults included in this study (1217 individuals in total) and compares the prevalence of these health events between migrant and non-migrant individuals. Migrant males under age 40 were more likely to have a connective tissue problem (e.g. back pain, chest pain, and joint pain) (*p* = 0.012), an infective or parasitic disease (*p* = 0.021) or a skin problem (*p* = 0.028) compared to non-migrant males under age 40. There was insufficient data available to test the difference in the prevalence of various morbidity categories between female migrants and non-migrants in either age group.

**Table 1:** Frequency of health problems among migrant and non-migrant adults (14–65 years) in southern India, 2013 (*n* = 1217)

	Prevalence among females (*n* = 581)				Prevalence among males (*n* = 636)				
	< 40 years		≥40 years		<40 years		≥ 40 years		
Disease Category	Prevalence among migrants (*n* = 13)	Prevalence among non-migrants (*n* = 395)	Prevalence among migrants (*n* = 4)	Prevalence among non-migrants (*n* = 169)	Prevalence among migrants (*n* = 174)	Prevalence among non-migrants (*n* = 250)	Prevalence among migrants (*n* = 14)	Prevalence among non-migrants (*n* = 198)	Overall prevalence (n = 1217)
Connective tissues	1 (7.69%)	15 (3.80%)	1 (25.0%)	31 (18.34%)	13 (7.47%)*	5 (2.0%)	2 (14.29%)	47 (23.74%)	115 (9.45%)
Nervous/sense organs	1 (7.69%)	13 (3.29%)	0	22 (13.02%)	6 (3.45%)	9 (3.60%)	1 (7.14%)	16 (8.08%)	68 (5.59%)
Circulatory/respiratory	0	1 (0.25%)	0	15 (8.88%)	4 (2.30%)	2 (0.80%)	3 (21.43%)	11 (5.56%)	36 (2.96%)
Digestive	1 (7.69%)	6 (1.52%)	0	6 (3.55%)	3 (1.72%)	7 (2.80%)	0	6 (3.30%)	29 (2.38%)
Injury/poisoning	0	0	0	2 (1.18%)	4 (2.30%)	4 (1.60%)	1 (7.14%)	7 (3.54%)	18 (1.48%)
Diabetes	0	0	0	4 (2.37%)	0	3 (1.20%)	0	10 (5.05%)	17 (1.40%)
Infective/parasitic	0	2 (0.51%)	0	1 (0.59%)	6 (3.45%)*	1 (0.40%)	0	2 (1.01%)	12 (0.99%)
Reproductive health	0	11 (2.78%)	0	1 (0.59%)	0	0	0	0	12 (0.99%)
Skin	0	4 (1.01%)	0	1 (0.59%)	4 (2.30%)*	0	2 (14.29%)	1 (0.51%)	12 (0.99%)
Ill-defined	0	2 (0.51%)	0	3 (1.78%)	0	0	0	0	5 (0.41%)
Genito-urinary	0	1 (0.25%)	0	0	0	0	0	0	1 (0.08%)
Totals	3 (23.08%)	55 (13.92%)	1 (25.0%)	86 (50.89%)	40 (22.99%)	31 (12.4%)	9 (64.29%)	100 (50.51%)	325 (26.71%)

*p < 0.05 based on Fisher’s exact test

### Health events ending or altering migration

Through the semi-structured interviews, several former migrants spoke of how their participation in migrant labour was detrimental to their health, forcing them to stop migrating altogether:

“I worked as a tailor in [an urban centre] six years ago, but I do not migrate anymore. That work is not suitable for my health. I stopped migrating because I was experiencing regular fevers and other health problems. I cannot work in the city. I will work in agriculture from now on” (45-year old male).

Additionally, one female interviewee spoke of how health problems forced her husband to stop working in construction:

“When my husband was working as a mason, he was getting worse pain in his shoulders and chest. He was also getting holes in his feet from the cement…He left the mason work and now tries to find work locally in agriculture” (35-year old female).

Some former migrants stopped migrating not because of the deterioration of their own health, but because of the poor health of a family member who accompanied them on their migration journey:

“Because of our son’s health problem, we cannot migrate for employment. We all worked together in a brick kiln cutting bricks near [an urban centre], but stopped nine years ago. Our son got very sick from the brick work so we had to return to our village. His health changed due to high fevers and bad headaches. He now has mental health problems and needs to be heavily medicated. We cannot leave our son alone. Someone has to take care of him in the house” (female and male couple).

Poor health from occupational or livelihood hazards experienced while migrating did not deter all migrants from continued future migration. Other interviewees opted to seek out alternative employment opportunities when they recognized the toll that their work was having on their health. One interviewee spoke of his son, who was in the process of looking for new work outside of their village:

“One year ago, my son went to work in an automotive manufacturing factory in [an urban centre]. He worked on the assembly line and had to stand all day. The work was not good for him so he left…He has submitted an application to be a police constable but has not heard anything. If he is not accepted as a police constable, he will go to [an urban centre] to work in another factory” (55-year old male).

### Perceptions of the determinants of internal migrant health

Of the 137 migrant households included in the quantitative part of this study, 62 (45.3%) saw an overall decline in the health of their migrant members as a result of their participation in labour migration. This decline in health was largely attributed to long working hours and physically demanding jobs. Conversely, 45 households (32.9%) saw no change in health and 18 households (13.1%) saw an overall improvement in the health of their migrant members. Enhanced mental health was the most frequently cited health improvement and was often attributed to good working hours and improved food security (Table 2).

**Table 2:** Perceptions of migrant member health from migrant households in southern India, 2013 (*n* = 137)

Perceived deterioration in health (*n* = 62)	
*How has health deteriorated for migrant members since migration started?*	*Total number of migrant households* *(% of all migrant households)*
Experienced a medium health problem (e.g. respiratory problem; broken bone; etc.)	29 (21.17%)
Body pain	18 (13.14%)
Decline in mental health	18 (13.14%)
Fever	11 (8.03%)
Decrease in energy level	10 (7.30%)
Chronic headache	10 (7.30%)
Major health problem (e.g. amputation; cancer; etc.)	5 (3.65%)
*Why has health deteriorated for migrant members?*	
Long working hours	24 (17.52%)
Job is physically demanding	24 (17.52%)
Poor working conditions and environment	14 (10.22%)
Bad employer	13 (9.49%)
Poor access to food	7 (5.11%)
Poor housing	5 (3.65%)
Started drinking	3 (2.19%)
Started drinking and smoking	1 (0.73%)
Long commute	1 (0.73%)
Unknown	1 (0.73%)
Perceived improvement in health (*n* = 18)	
*How has health improved for migrant members since migration started?*	*Total number of migrant households* *(% of all migrant households)*
Improvement in mental health	17 (12.41%)
Increased energy level	8 (5.84%)
Acquired new skills	1 (0.73%)
*Why has health improved for migrant members?*	
Good working hours	13 (9.49%)
Good access to food	10 (7.30%)
Job is not physically demanding	9 (6.57%)
Good employer	6 (4.38%)
Good housing	3 (2.19%)
No perceived change in health (*n* = 45)	
Unknown (*n* = 12)	

Respondents could report multiple health outcomes and reasons for health outcomes

Consistent with the findings from the surveys, the semi-structured interviews revealed mixed results related to migrant experiences with health. Interviewees from migrant households commonly expressed their concerns for the health and wellbeing of their migrant members who were currently engaged in migrant labour. In particular, interviewees were wary of poor working conditions and how these conditions might contribute to adverse health outcomes and subsequently decreased earning potential for their migrant family members. Furthermore, and consistent with survey findings, poor health outcomes were most often linked to jobs that required heavy manual labour or long working hours.

Connective tissue problems was the most commonly discussed health event among migrant households, as this was seen to directly interfere with or impede further manual work. In cases where migrants were paid based on work completed, physical pain was framed in terms of impacting not only the health of the migrant, but also the financial stability of the household. However, it was noted that some migrant work that involved manual labour was often no more strenuous or physically demanding than local agricultural work. In comparing migrant work with local agricultural work, one interviewee argued that agricultural work in his village was more physically demanding:

“I have worked in the city. After a day of work in the city, I would need to sleep for a day in order to recover from the work. But now that I am doing heavy agricultural work in the field, I need longer to recover from that work. People who go to work outside of the village are no longer suited for agricultural work” (47-year old male).

In addition, changes in the mental health and personality of migrant members were frequently noted as an outcome of participation in migrant labour. The nature of these changes was primarily tied to the type of work that migrants were engaged in, but was also connected to factors outside of the workplace. Demanding work conditions and hard manual labour were generally associated with a decline in mental health or a ‘dull’ personality. One interviewee noted the difficult trade-off between a perceived decline in the mental health in his sons and the necessity of income they generated for their household:

“I am seeing lots of changes in my sons. If my sons stayed here, it means they have no work to do, but they are energetic and their personality is good. When they come back from work in the city, they are so tired, and their personality is dull and weak. Every day, they carry cement bags from the ground floor to the sixth floor. They are also facing lots of problems like shelter, sleep, and sometimes problems getting food. I would like to keep my sons in my home, but we need the income” (45-year old male).

Conversely, some households saw their migrant members grow personally and professionally by taking on new opportunities outside of their village:

“Our middle son is a very talented person, and has learned a lot as a result of going to the city for work. Before, in the village, he had a dull personality and hated doing work in the field. Now, in the city, he is using his talents” (64-year old male).

In addition to working conditions, living arrangements including housing, access to adequate food and clean water, access to medical care, and the physical environment were considered to influence the health of migrants. Experiences with each of these factors differed depending on destination, industry, and the presence and strength of social networks. However, there was a general perception that in most cases, food was more readily available in the city than in the village, which contributed to enhanced migrant wellbeing:

“My son is healthy in the city. He is eating three times per day and earning a good income. If he were to stay in the village, he would only eat two times per day. He would also have to do agricultural work in the village, which would make him unhealthy” (50-year old male).

Furthermore, social networks were seen to mediate some of the challenges associated with migrating to a city including securing employment and housing. In particular, there were multiple instances of groups of young men from a particular village migrating together to the same destination to work for the same employer. Alternatively, several individuals would establish a relationship with a particular employer and then send for family members or friends. However, these networks were also viewed by a minority of migrant households as a source of ‘bad habits’ including excessive alcohol consumption and smoking. There were also a few interviewees who discussed how their migrant member did not migrate with family or friends and worked alone to secure employment.

A few survey respondents (*n* = 12) acknowledged that they were unaware of the health of their migrant household members. One reason for this lack of knowledge was attributed by interviewees to migrants obscuring their health problems from their family members:

“I know my sons are facing lots of health problems like frequent colds, coughs, and high fevers. But they don’t share these problems with us because they do not want us to worry. They go to clinics on their own and take care of their own health problems” (45-year old male).

“If our son stays in the village, he is healthy. When he migrates, we do not know what health problems he faces in the city. All we know is that mason work is difficult work to do” (female and male couple).

### Perceptions of migrant health from non-migrant households

There was consensus among interviewees who did not have any migrant family members that migrant workers and migrant households appeared ‘well-off’ and ‘healthy.’ In these cases, health was synonymous with perceived financial wellbeing and stability. A number of non-migrant households mentioned how they either aspired to be like migrant households or desired for members of their family to migrate for work:

“When I see people who have migrated return to the village, they appear to look very good and live in good conditions. I wish my sons could migrate for work. If they did, we could live like the migrant families and earn a good income” (70-year old male).

Interviewees from non-migrant households also perceived the physical environment as a threat to migrant health and contrasted the quality of the environment in the village to the quality of the environment in the city. In particular, the apparent poor air and water quality in the city was viewed as a potential source of health problems for migrant workers among non-migrant households.

## Discussion

### Exploring the ‘healthy migrant effect’

Using a mixed methods approach, our findings reinforce the notion that internal labour migration is a selective process. As other studies have demonstrated, health considerations can shape pre-migration decisions and internal migration trajectories [26, 35, 43]. However, our findings also show that the selectivity of internal labour migration processes is not necessarily synonymous with the ‘healthy migrant effect.’

Our quantitative data on self-reported illness demonstrates that migrant workers and non-migrant adults from the same rural area have similar health profiles. However, migrant males under age 40 appear to have a higher prevalence of some health problems including connective tissue problems. Moreover, for health problems reported among migrant workers, the relationship between a particular health outcome and migrant labour activities was obvious in some cases (e.g., a broken arm due to a workplace accident). At other times though, the association between health and migrant labour was less clear (e.g., joint pain attributed to ongoing manual labour), however the causal relationship between migration and a poor health outcome was clear in the mind of the respondent.

Despite these connections between internal migrant activities and poor health outcomes, the similar health profile between migrant and non-migrant adults in this context calls into question the idea that internal migrant workers have a distinct health advantage over their non-migrant counterparts. In the research area, non-migrant adults are largely engaged in agricultural work, either on their own land, or on the land of large landowners. This work is physically demanding, and as other studies have demonstrated, rural agricultural workers, are at risk for a host of occupational hazards and health problems [44, 45]. In some cases, rural-to-urban and rural-to-rural migrant workers are exposed to similar working conditions as non-migrant workers.

This reality may have several implications for understanding the similar health profiles across migrant and non-migrant individuals in addition to the construction of the ‘healthy migrant effect’ in this context. First, it is possible that the ‘healthy migrant effect’ operates as it is theorized to, and that healthy migrants lose their supposed health advantage over non-migrant individuals, leading to an eventual equalizing of health across individuals from this area. This explanation is supported by the majority of migrant households who believed that the health of their migrant members declined as a result of their participation in migrant labour. Second, as a result of the similarities in working conditions across migrant and non-migrant industries, it is also possible that any generalized changes in health in migrant populations are mirrored by non-migrant populations. Finally, and as demonstrated by other studies, internal migrant worker status is fluid [46]. Of particular importance to this context, the high proportion of temporary labour migration means that many individuals included in this study balance working locally and in another destination, which makes it difficult to attribute a specific health problem exclusively to migrant labour activities.

A combination of these explanations likely provides insights into the comparable health profile across migrant and non-migrant individuals in this context. These findings also underscore the problem with generalizing the health statuses of migrant workers or how the health of migrant workers will be impacted as a result of their participation in migrant labour. At the same time, it is important to recognize the unique hazards that migrant workers may face as a result of potentially exploitative labour arrangements or insecure living conditions. Indeed, several interviewees detailed how their migrant work experience or the work experience of a family members was directly connected to poor and often serious health outcomes. Thus, and as detailed below, there is need to understand the conditions under which internal labour migration can negatively influence health outcomes.

### The determinants of internal migrant health

The health of migrant workers is mediated through a number of pathways, which directly influence health outcomes. However, the emphasis on health outcomes associated with internal labour migration can, at times, neglect the causal pathways that impact the health of migrant workers. Instead, internal labour migration is often viewed as the primary exposure variable whereby negative changes in migrant health are associated with their vulnerability [21]. The prioritization of health outcomes correlated with internal labour migration over the determinants of internal migrant health, contributes to an incomplete understanding of how internal migration processes and migrant labour shape health in a particular context. Furthermore, this limited understanding and measurement of the determinants of internal migrant health can lead to a lack of or misguided public health interventions aimed at addressing these determinants.

In our study, participants identified two broad categories that shaped internal migrant experiences with health. First, occupational factors including industry, position, working hours, working conditions, and employer, were considered to have a significant impact on the health of migrant workers. For example, positions with heavy manual labour components and long working hours were seen as detrimental to physical and mental health [17–19]. Conversely, positions where migrants were given challenging tasks with good working hours were viewed as beneficial for mental health. Thus, there is a need to look beyond industries to the diverse positions that migrants hold, as well as the reward mechanisms within industries, to understand how these various positions within a specific industry may differentially influence health [19, 47].

Second, livelihood factors including destination, housing, food security, water quality, access to medical care, social networks, and the physical environment were viewed to impact migrant worker health. Beyond these factors identified by interviewees, inadequate access to sanitation in combination with a lack of safety, abuse, and police harassment can be additional determinants of internal migrant worker health [3]. Although not explicitly referenced by interviewees, it was implicitly demonstrated that gender shaped experiences with migration and subsequently health [15, 18, 48–51]. In this setting, internal labour migration was a male-dominated activity, although there was evidence of a few females migrating, often with their male counterpart. It is important to consider how gender can shape vulnerability, exploitation, and agency within the workplace and how these experiences influence the economic, social, and health-related outcomes associated with labour migration [48]. For example, in some industries, there is evidence of differential wage rates between women and men, with women paid less for the same work completed [52].

Like occupational factors, respondents indicated that the nature of the relationship between individual livelihood factors and migrant health was complex and dependent on context. For example, the presence and strength of social networks were viewed by the majority of respondents as beneficial to the health of migrant workers as these networks could assist in securing employment and housing [53, 54]. In particular, these networks allowed migrant workers to collectively address the occupational and livelihood factors outlined above. The importance of social networks in securing housing has important implications for migrant health as migrants often live in insecure or temporary accommodations with risk of eviction [2, 3]. However, social networks, as well as the influences of urban lifestyles for urban migrants, were also seen to be detrimental to health when ‘bad habits’ such as excessive alcohol consumption and smoking were promoted [55, 56].

This examination of the occupational and livelihood factors that influenced migrant health in this context provides several insights. First, greater focus needs to be on identifying the occupational and livelihood factors that operate within internal labour migration processes and are responsible for shaping health [17]. We demonstrated that village-based mixed method studies can be an important starting point for identifying and defining what these factors are and how they may function within a particular migration trajectory. In addition, bringing the perspectives of migrant and non-migrant households together aids our understanding of how these groups perceive and experience the determinants of internal migrant health. Second, following identification, these factors need to be measured using both quantitative and qualitative methods to better understand the extent to which they influence internal migrant health [57, 58]. In this way, we can begin to examine, for example, how migrant experiences in specific positions within certain industries may impact a particular health outcome. Finally, a broader definition of health is needed that moves beyond a focus on health as the absence of physical disease [59], and recognizes that health encompasses an individual’s ability to adapt and manage in a new setting [60]. This understanding allows us to better assess whether or not internal migrants are able to fully leverage the purported benefits of internal migration for themselves and their households.

### Limitations

The use of self-reporting in addition to low health literacy in this context may have led to underreporting of morbidity among both migrant and non-migrant households. However, self-reports provide meaningful insights into the burden of disease in low-resource settings and offer a baseline for further investigation in particular morbidity categories [61]. The cross-sectional nature of this study meant that we were only able to examine migrant and non-migrant health statuses at a point in time, rather than over a longer time frame. Additionally, as a result of the migration patterns and migrant labour arrangements present in this setting, some of the experience with labour migration and the subsequent influence on health and well-being may only be applicable to migrants and migrant households from the area where the study was conducted. However, these findings provide an important case study that can be compared to other similar settings across India.

## Conclusions

Using an exploratory mixed methods study conducted in 26 villages in the Krishnagiri district of Tamil Nadu, we explored how the ‘healthy migrant effect’ operates in this context. In addition, we identified occupational and livelihood factors that were viewed as critical determinants of internal migrant health and examined how these determinants influence the health of migrant workers in this setting. We showed how individual determinants can have differential impacts on health outcomes and subsequently cautioned against generalizations of migrant worker experiences with health. The importance of internal labour migration for individuals and households in this setting and throughout India means the diagnosis of health problems associated with internal labour migration must be combined with the identification and measurement of the determinants of internal migrant health. With greater resources allocated to public health interventions that respond to these contextual determinants of health, it is more likely that internal labour migration will contribute to the expected gains in human development for migrant workers and their households.

## Footnotes

1 Based on a rural poverty line of 27 Indian Rupees (INR) per day as defined by the Indian Planning Commission in 2011.
